# Anti-GPI scFv as a Promising Tool for Intervention Against Cerebral Malaria in Mice

**DOI:** 10.3390/ijms27072950

**Published:** 2026-03-24

**Authors:** Sandra Gabriela Klein, Kelem Cristina Pereira Mota, Bruna Cristina Borges, Mylla Spirandelli Vieira, Matheus Morais Neves, Ludmilla Silva Mendes, Flávia Batista Ferreira, Isabela Lemos de Lima, Fabiana de Almeida Araújo Santos, Luciana Machado Bastos, Wânia Rezende Lima, Luiz Ricardo Goulart Filho, Murilo Vieira da Silva

**Affiliations:** 1 Biotechnology Laboratory in Experimental Models—LABME, Federal University of Uberlândia, Uberlândia 38402-018, MG, Brazil; klein.sandra@ufu.br (S.G.K.); myllaspirandelli@gmail.com (M.S.V.); matheusmoraisneves@gmail.com (M.M.N.); isabela.lemos@ufu.br (I.L.d.L.); waniarezende@ufcat.edu.br (W.R.L.); 2 Laboratory of Nanobiotechnology (NANOs), Federal University of Uberlândia, Uberlândia 38402-018, MG, Brazil; 3 Institute of Biotechnology, Federal University of Catalão, Catalão 75706-881, GO, Brazil

**Keywords:** nanobiotechnology, *Plasmodium berghei* ANKA, glycosylphosphatidylinositol, single-chain variable fragment, experimental cerebral malaria

## Abstract

Malaria remains a major global health challenge. While treatments targeting parasite replication exist, effective interventions for neurological manifestations are scarce, necessitating new strategies for cerebral malaria. In this study, we investigated the effect of a single-chain variable fragment (scFv) against glycosylphosphatidylinositol (GPI) as an intervention tool to mitigate the effects of *Plasmodium* in a preclinical model. We used C57BL/6J mice infected with *Plasmodium berghei-*ANKA (PbA) and treated them with anti-GPI scFv or phosphate-buffered saline (PBS) on days 0, 3, and 6 post-infection. Uninfected controls were treated on the same days with scFv or PBS. The animals were evaluated for morbidity and mortality, body weight, parasitemia, blood count, cytokines, and histopathology. Results show that anti-GPI scFv prevented lethality in 71.4% of infected animals and promoted recovery from weight loss. Furthermore, the intervention inhibited neurological and systemic signs, reduced parasitemia, and improved hematological and histopathological parameters in the brain, lungs, and kidneys. In conclusion, anti-GPI scFv exerts a significant systemic effect on experimental cerebral malaria (ECM) pathology, representing a promising tool for severe manifestations of the disease.

## 1. Introduction

Malaria is a globally distributed and potentially fatal parasitic disease [1,2]. According to estimates from the World Health Organization in 2023, there were 263 million cases and 597,000 deaths due to malaria, with the African region accounting for 94% of cases and 95% of deaths [1]. The most vulnerable groups include children under 5 years old, pregnant women, and patients with HIV (Human Immunodeficiency Virus) or other immunosuppressive conditions [1,3,4].

Caused by protozoa of the *Plasmodium* genus and transmitted by the *Anopheles* mosquito, malaria has five species capable of causing diseases in humans [1,5]. However, *P. falciparum*, *P. vivax*, and *P. malariae* are epidemiologically more significant, with *P. falciparum* predominant in Africa and Asia, and *P. vivax* in the Americas [1,2].

When infected, individuals may be asymptomatic or present symptoms ranging from mild to severe [6]. Malaria is classified as uncomplicated, where patients exhibit mild to moderate symptoms such as headaches, muscle aches, fever, and lethargy, and complicated, with severe symptoms including severe anemia, metabolic acidosis, and neurological symptoms [6,7].

Current malaria treatment is based on antiparasitic drugs such as chloroquine, artemisinin and artesunate, supplemented by supportive care as needed [8]. However, resistance to antiparasitic drugs, particularly artemisinin and combination therapies, is a growing concern, particularly in Southeast Asia [7,9]. *P. falciparum* has shown resistance to all antiparasitic drugs used in Africa, Asia and South America, while *P. vivax* has shown resistance to chloroquine in Papua New Guinea and Indonesia [10,11]. This highlights the difficulties and limitations in treating malaria and underscores the need for new drugs.

Despite these difficulties with drugs, there are currently two vaccines approved by the WHO and in programmatic use [12]. Both are *P. falciparum* Circumsporozoite Protein (CSP) subunit vaccines and are recommended for young children in endemic countries in Africa. The first is RTS, S/AS01 (Mosquirix), already integrated into national programs in several African countries, which reduces cases of clinical malaria with 40 to 55% efficacy over 1 to 3 years, requiring four doses [13,14]. The second is R21/Matrix-M, with 70–75% efficacy over 12 months and the advantage of being cheaper and easier to produce on a large scale [13,14]. In addition to these, there are other vaccines in development, targeting, for example, the blood stage or placental malaria [12]. However, other interventions and treatments are still needed to combat the disease [15].

In this context, new drugs and therapeutic targets are essential. Our group has studied glycosylphosphatidylinositol (GPI), a molecule recognized as a pathogen-associated molecular pattern (PAMP) that triggers the production of fundamental pro-inflammatory cytokines in the pathogenesis of cerebral malaria [16,17]. PAMPs play a critical role in parasite adhesion to host cells and disruption of the blood–brain barrier, which is a key factor in the development of cerebral malaria [18]. Recent studies have shown that GPI anchors, a major PAMP of *Plasmodium*, activate innate immune receptors such as TLRs, leading to the production of pro-inflammatory cytokines and endothelial dysfunction [19,20]. These findings suggest that targeting GPI may not only inhibit parasite adhesion but also prevent blood–brain barrier breakdown, providing a dual therapeutic approach [21,22].

Although eukaryotic cells generally display GPI on their surface, there is a structural difference between *P. falciparum* and human GPI, making parasite GPI a promising target for new drug development [19,22].

The rationale behind using the PbA-infected mouse model as a preclinical tool for validating the anti-GPI molecule, which was initially raised against *P. falciparum* GPI, requires justification, especially considering the phylogenetic and biological differences between these two *Plasmodium* species. Even though these differences exist, the literature on which this work is based and grounded indicates that there is increasing biochemical data pointing towards the high degree of conservation in GPI biosynthesis and structures in the *Plasmodium* genus [22,23].

Early studies have shown the basic glycan core of Plasmodium GPI structures, as well as the high degree of conservation in GPI biosynthesis, including the stepwise addition of mannose units and the lipid moiety, in the *Plasmodium* genus [22,23]. This high degree of conservation in GPI structures points towards the possibility of the anti-GPI single-chain variable fragment (scFv) raised against *P. falciparum* GPI being able to recognize epitopes present on PbA GPI, thus justifying the translational potential of the murine model. Further, the experimental cerebral malaria (ECM) model, which utilizes PbA-infected C57BL/6 mice, is the most used preclinical model for validating anti-malarial therapeutics targeting human cerebral malaria caused by *P. falciparum* [16,17]. This is because there are several pathophysiological features common to these two conditions, including neurological manifestations, blood–brain barrier compromise, infected erythrocyte sequestration, and high levels of pro-inflammatory cytokines, which are known to be mediated through GPI-induced TLR activation [19,20].

Therefore, our hypothesis is that an anti-*P. falciparum* GPI antibody fragment may be promising against the exacerbation of malaria cases, and our goal is to test a selected anti-scFv (single-chain variable fragment) against *P. falciparum* GPI in ECM.

## 2. Results

### 2.1. The scFv Increases Survival and Restores Body Weight in Mice Infected with PbA

**Figure 1:**
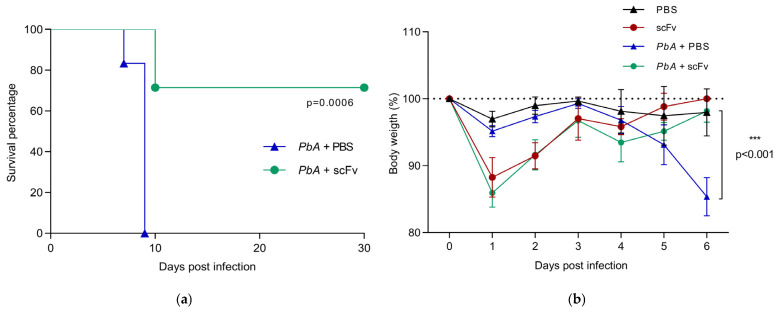
(**a**) Survival curve, demonstrating that scFv increases the survival of mice infected with PbA. PbA+PBS group (*n*:6), PbA+scFv group (*n*:7). (**b**) Percentage of body weight gain/loss in C57BL/6 mice infected/non-infected with PbA, treated/untreated with scFv. Groups: PBS (*n* = 3), scFv (*n* = 3), PbA+PBS (*n* = 6), PbA+scFv (*n* = 6). (*** *p* < 0.001). For the survival curve, the Kaplan–Meier test followed by the Log-rank test (Mantel–Cox) was utilized, while for body weight, Student’s *t*-test was conducted.

In the ECM model, the progressive loss of vital signs associated with coma, microhemorrhage, and weight loss is a typical feature of cerebral malaria in C57BL/6 mice infected with PbA. To investigate whether the scFv could influence the ECM condition, the animals were intraperitoneally infected with 1 × 10^5^ PbA-infected red blood cells and treated with 100 µg/animal of scFv at the time of infection. Subsequently, on the third and sixth days post-infection (dpi), the animals received additional scFv treatment. Mortality data were recorded throughout the infection and treatment periods (Figure 1a). The results of animals infected but not treated with scFv, only with PBS, were also collected and used as a control (Figure 1). The survival curve revealed that animals untreated with scFv succumbed between the seventh and ninth dpi, while those in the scFv-treated group started dying on day 10, with most animals succumbing at day 30 dpi. More specifically, the survival curve showed that in the PBS-treated group, one animal died on day 7, and five animals died on day 9 post-infection, whereas in the scFv-treated group, two animals died on day 10, and five animals died on day 30 post-infection. Thus, animals treated with 100 µg of scFv were significantly more resistant to PbA infection compared to scFv-untreated and PbA-infected animals (*p* < 0.0006). Furthermore, in monitoring survival (Figure 1a), it was observed that all animals in the PBS-treated group infected with PbA succumbed between the seventh and ninth day post-infection, presenting clinical signs of cerebral malaria. In contrast, unlike the previous group, infected animals and the scFv-treated group remained alive and free of clinical manifestations of cerebral malaria until the 30th dpi, except for two animals that died on the 10th dpi.

As mentioned above, it is well-known that weight loss is common in the ECM model. For this reason, during the experiments, the effect of scFv per se was investigated in both non-infected and PbA-infected animals. To determine the morbidity curve, four groups of animals were observed daily (Figure 1b). Morbidity curve illustrates the body weight monitoring, where all animals showed an initial weight reduction. From the third dpi, there was a progressive weight reduction in the PbA+PBS-treated group until the sixth dpi. In contrast, the scFv-treated group and PbA-infected animals exhibited body weight gain on days 2, 3, 5, and 6 post-infection. Notably, the PBS-treated group and PbA-infected animals on the sixth dpi does not recover their weight and exhibited an average weight loss of approximately 12% (88.27 ± 8.7; *** *p* < 0.0091) compared to the scFv-treated group (96.97 ± 7.5; Figure 1b). Taken together these results showed that treatment with scFv minimizes the symptoms of ECM and increases the survival of animals infected with PbA.

We observed a marked reduction in body weight in the scFv-treated groups shortly after the first administration (day 0), including both uninfected (scFv) and infected (PbA+scFv) animals. Since treatment and infection were performed on the same day, it is possible that the initial weight loss reflects a transient acute response to scFv administration rather than an effect of infection. Although we did not perform additional metabolic or behavioral assessments at this initial point, we cannot rule out the possibility of a temporary reduction in food and water intake resulting from an acute systemic response.

It is important to note that this weight loss was transient. Animals in both scFv-treated groups recovered in the following days. A similar, albeit less pronounced, reduction was also observed after the second administration on day 3, again followed by recovery. In contrast, the PbA+PBS group showed a progressive decline in body weight after day 4, without recovery, which is consistent with disease progression.

Therefore, although scFv administration may be associated with a short-term physiological response reflected by transient weight loss, it did not result in sustained clinical worsening. The pattern of recovery observed in the PbA+scFv group contrasts with the continuous decline observed in the PbA+PBS group, reinforcing the protective effect of treatment throughout the course of infection.

### 2.2. The scFv Progressively Reduces the Parasitic Burden in Mice Infected with PbA

**Figure 2:**
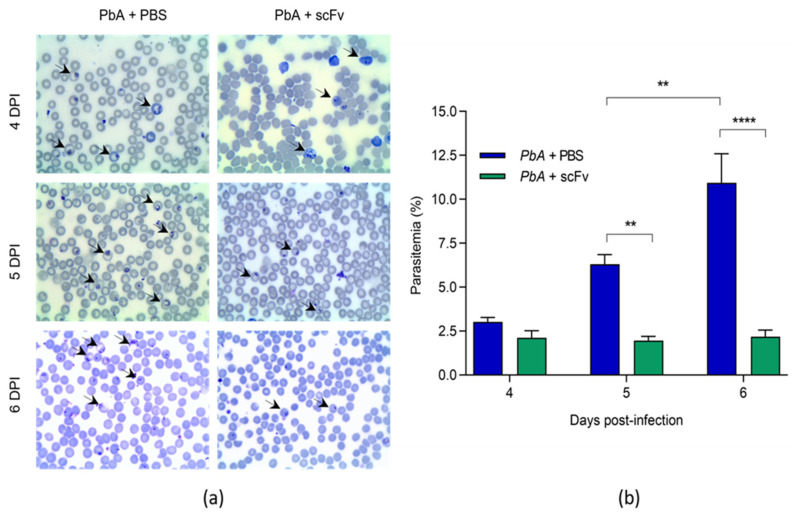
Parasitemia in scFv-treated and untreated anti-GPI mice infected with *P. falciparum.* In (**a**) illustrative image of optical microscopy showing the progression of parasitemia between the 4th and 6th dpi (magnification 100×). Arrows indicate the *Plasmodium* inside the cells (**b**) Percentage of parasitemia over days 4, 5, and 6 post-infection. In (**b**), statistically significant differences can be observed between the PbA+PBS and PbA+scFv groups on days 5 and 6 post-infection. Parasitemia in the PbA+scFv group remained stable, with no statistically significant differences when comparisons were made within the same group over the course of the infection. The infecting dose was 1 × 10^6^ infected erythrocytes per mouse. The graph shows mean and standard deviation, with groups of *n* = 6. (** *p* < 0.005; **** *p* < 0.0001). A two-way ANOVA was conducted for parasitemia, followed by Bonferroni’s multiple comparison test.

Through the count of infected/non-infected erythrocytes in blood smears, the percentage of parasitemia was analyzed on the 4th, 5th, and 6th days post-infection (dpi) (Figure 2b). The PbA+PBS group showed a progressive increase in parasitemia, as indicated by the arrows in Figure 2a over the days, with 3.02 ± 0.60 on the 4th dpi, 6.30 ± 1.34 on the fifth dpi, and 10.93 ± 4.05 on the 6th dpi. In the PbA+scFv group, parasitemia remained stable over the days, with 2.11 ± 0.97 (4th dpi), 1.95 ± 0.59 (5th dpi), and 2.18 ± 0.91 (6th dpi), demonstrating that parasitic replication was controlled in the presence of scFv treatment. The mice in the PbA+scFv group exhibited significantly lower parasitemia compared to the control group on the last two days of analysis, with an average difference between the groups of 4.35% (** *p* < 0.0050) on the 5th dpi and 8.75% (**** *p* < 0.0001) on the 6th dpi. These results indicate that scFv plays a crucial role in reducing parasitemia caused by PbA.

### 2.3. The Treatment with scFv Reduces Clinical Signs of Cerebral Malaria and Improves the Overall Clinical Condition of Mice Infected with PbA

**Figure 3:**
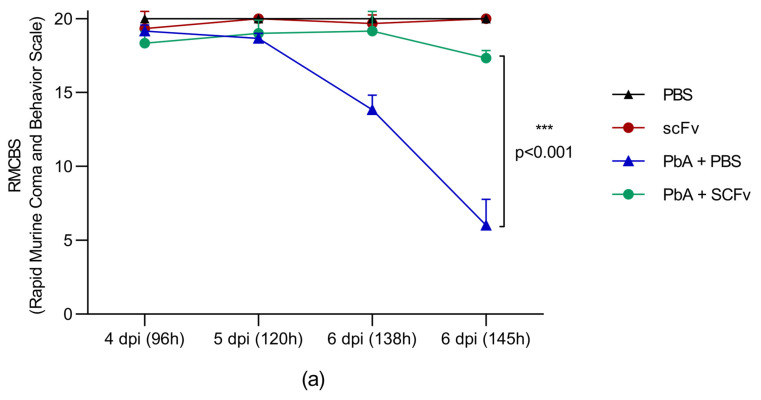

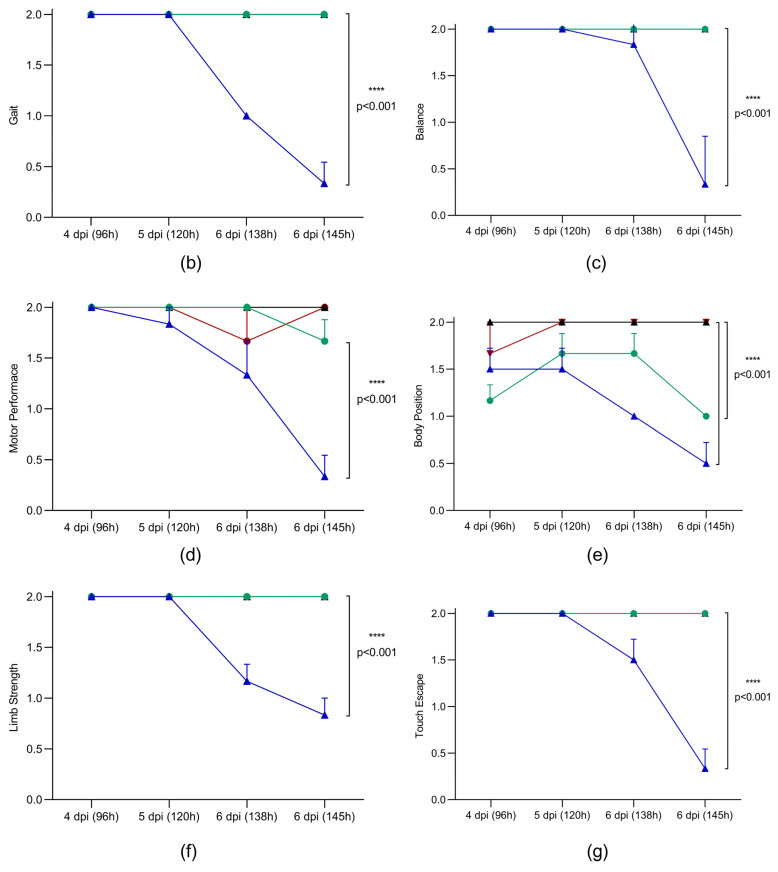

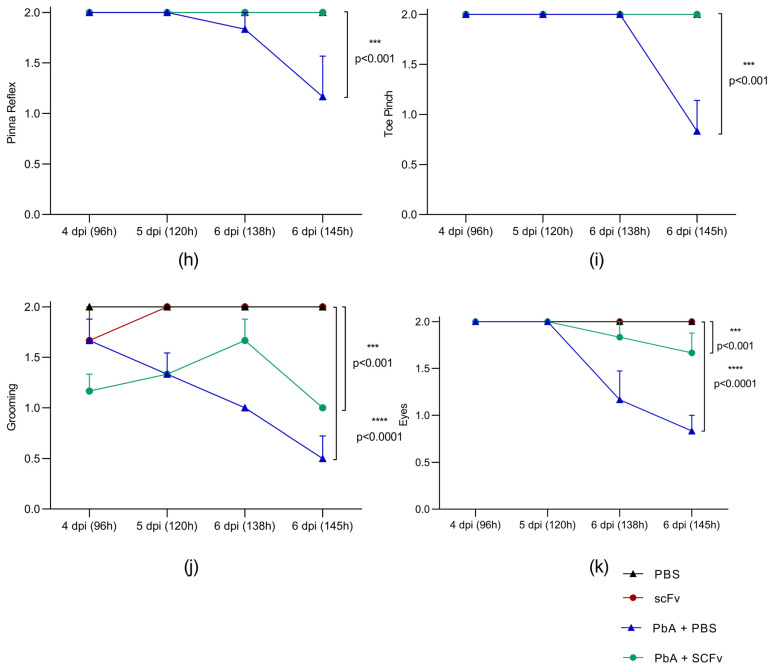
Morbidity score (Rapid Murine Coma and Behavior Scale). The graph (**a**) presents the sum of scores, and the other graphs represent each of the analyzed parameters individually. From (**b**–**k**) individual assessment parameters used to construct the behavioral score, in order: gait, balance, motor performance, body position, limb strength, touch escape, pinna reflex, toe pinch, grooming and eyes. Groups: PBS (*n* = 3), scFv (*n* = 3), PbA+PBS (*n* = 6), PbA+scFv (*n* = 6). The graphs show mean and standard deviation (*** *p* < 0.001; **** *p* < 0.0001). The statistical analysis was performed using two-way ANOVA followed by Tukey’s multiple comparison test.

As the progressive loss of cognitive and motor performance has been associated with brain damage due to severe malaria, it was tested whether treatment with scFv could also reveal changes in the RMCBS score in malaria-infected mice compared to untreated animals. Thus, RMCBS score values were assigned at four timepoints, as shown in Figure 3. For the uninfected groups, PBS-treated (*n* = 3) and scFv-treated (*n* = 3), it is notable that all cognitive and motor parameters, such as gait, balance, motor performance, body position, limb strength, touch escape, pinna reflex, toe pinch, grooming, and eyes, were preserved (Figure 3b–k). Conversely, the untreated PbA-infected group showed the onset of RMCBS score decline (Figure 3) starting at day 5 post-infection (dpi), with a sharp decrease in the RMCBS score observed by 138 h. The average RMCBS score was 13.87 compared to approximately 19.17 in the scFv-treated group (**** *p* < 0.0001). The difference became even more pronounced at 145 h post-infection, with a cognitive and motor performance score averaging 6 in the untreated PbA-infected group (*n* = 6), compared to 17.33 in the scFv-treated group (*n* = 6) (**** *p* < 0.0001). Notably, when individual parameters were analyzed, scFv treatment showed the greatest protective effect on gait, balance, limb strength, touch escape, pinna reflex, and toe pinch (Figure 3c,f–i, respectively). Moreover, it was observed that the PbA-infected and scFv-treated group maintained a significantly lower score than the uninfected groups in the parameters of body position, grooming, and eyes (Figure 3e,f,k, respectively).

In addition, in the evaluations of each score, the PbA-infected and scFv-treated group performed better than the PbA-infected and PBS-treated group, except for grooming, where there was no statistical difference between the groups.

### 2.4. The scFv Treatment Improves Thrombocytopenia and Monocytopenia in Mice Infected with PbA

**Figure 4:**
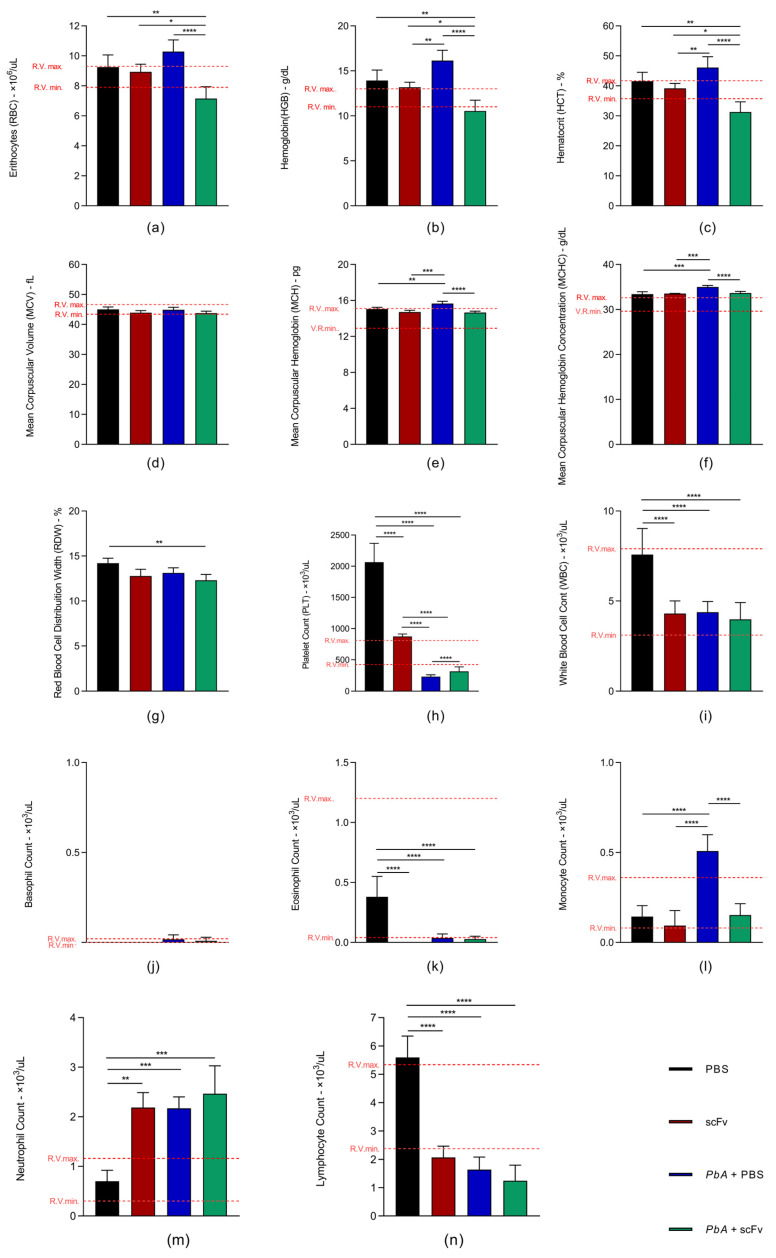
The graphs depict hematological parameters of C57BL/6J mice infected and uninfected with PbA, treated with scFv or PBS. From (**a**–**g**), parameters of the red blood cell series are presented, (**h**) platelet count, and from (**i**–**n**), parameters of the white blood cell series are presented. Reference values were based on the same animal model and age from The Jackson Laboratory (JAX^®^). Groups: PBS (*n* = 3), scFv (*n* = 3), PbA+PBS (*n* = 6), PbA+scFv (*n* = 6). Mean and standard deviation are represented (* *p* < 0.05; ** *p*< 0.01; *** *p* < 0.001; **** *p* < 0.0001). The statistical analysis was performed using two-way ANOVA followed by Tukey’s multiple comparison test.

The animals in the PbA+PBS group showed polycythemia, evidenced by increased red blood cell count (Figure 4a) and hematocrit (Figure 4c), accompanied by elevated hemoglobin levels (Figure 4b). Although anemia is common in malaria cases, no obvious anemia was observed due to severe dehydration on the day of collection, resulting in reduced circulating fluid volume and consequently increased red blood cell concentration. The red cell indices showed normal MCV (Figure 4e), increased MCHC (Figure 4f) indicative of possible hemolysis, and increased RDW (Figure 4g). Additionally, thrombocytopenia (Figure 4h), leukopenia (Figure 4i) due to lymphocytopenia (Figure 4n) and eosinopenia (Figure 4k), with normal basophil counts (Figure 4j), neutrophilia (Figure 4m) with a right shift, and monocytosis (Figure 4l) were observed.

In the PbA+scFv group, normocytic normochromic anemia (Figure 4c,d,f), thrombocytopenia (Figure 4h), leukopenia (Figure 4i) due to lymphocytopenia (Figure 4n) and eosinopenia (Figure 4k), and neutrophilia (Figure 4m) with a right shift were observed. These results are typical of an acute response, considering the consumption of leukocytes associated with an increase in neutrophils without signs of regeneration.

The decrease in erythrocytes, hemoglobin, and hematocrit observed in the PbA+scFv group is consistent with infection-associated anemia, since these animals still had parasitemia at 6 dpi (2.18% ± 0.91). Therefore, some degree of anemia was expected.

It is important to note that, although malaria is typically associated with anemia, the PbA+PBS group had comparatively higher erythrocyte indices at 6 dpi. However, this group exhibited marked clinical decline, including reduced motor performance, altered body posture, and neurological signs related to lower water intake, as well as apparent dehydration at the time of blood collection, suggested by decreased skin turgor and increased blood viscosity. These findings are consistent with hemoconcentration due to dehydration, which may have masked the anemia expected in this group.

Thus, we interpret the lower erythrocyte indices in the PbA+scFv group as a reflection of anemia associated with infection, without evidence of hemoconcentration, while the elevated values in the PbA+PBS group likely reflect a reduction in plasma volume related to dehydration, rather than actual preservation of erythrocyte mass.

### 2.5. The Treatment with scFv Stimulates the Production of Pro-Inflammatory Cytokines in the Liver and Lung of Animals Infected with PbA

**Figure 5:**
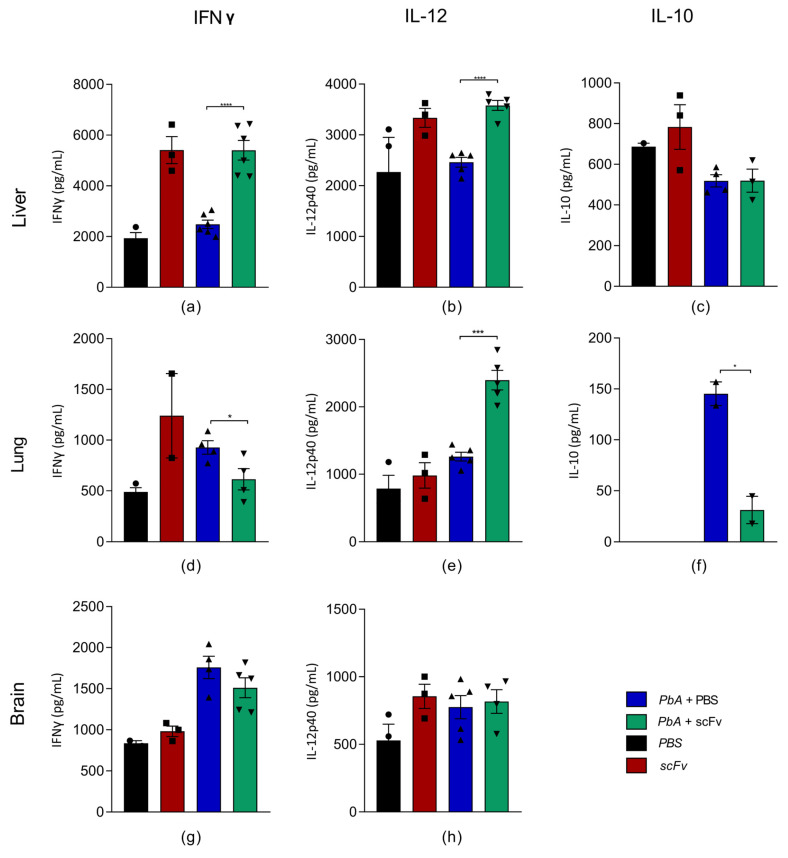
Cytokine panel of C57BL/6J mice infected or not with PbA and treated or not with scFv anti-GPI from *P. falciparum*. Cytokines were measured by ELISA in liver, lung, and brain homogenates. In the liver, IFN-γ (**a**), IL-12p40 (**b**), and IL-10 (**c**) were measured; in the lungs, IFN-γ (**d**), IL-12p40 (**e**), and IL-10 (**f**) were measured; and in the brain, IFN-γ (**g**) and IL-12p40 (**h**) were measured. Groups: PBS (*n* = 3), scFv (*n* = 3), PbA+PBS (*n* = 6), PbA+scFv (*n* = 6). Circles, squares, triangles and inverted triangles represent, respectively, the animals that make up each group and their individual data. The graphs represent mean and standard error of the mean (* *p* < 0.05; *** *p* < 0.001; **** *p* < 0.0001). The statistical analysis was conducted using two-way ANOVA followed by multiple comparisons tests of Holm–Sidak and Tukey.

Treatment with scFv showed a significant impact on the modulation of the immune response in different tissues. In the liver, we observed an increase in the production of IL-12p40 (Figure 5b) and IFNγ (Figure 5a), cytokines known to promote a pro-inflammatory immune response and activate Th1 cells. This profile suggests that scFv may be stimulating a more robust immune response against infection in this tissue.

In the lungs, treatment with scFv resulted in an increase in IL-12p40 production (Figure 5e), a reduction in both IFN-γ (Figure 5d) and IL-10 (Figure 5f). This pattern suggests a modulated pro-inflammatory response. This balanced cytokine profile may facilitate effective parasite control while preventing excessive inflammation-mediated lung injury in infected animals.

In the brain, the levels of IL-12p40 (Figure 5h) and IFNγ (Figure 5g) remained close to those of the infected control group, suggesting that scFv may not be modulating the immune response in this tissue in the same way as in the others, which is positive, as exacerbation of the immune response in the brain would bring consequences related to cerebral malaria. Unfortunately, the impossibility of quantifying IL-10 in the brain due to the loss of samples limited our ability to fully interpret the immune response at this site, as an increase in the IL-10 response could explain the absence of clinical signs related to cerebral malaria in the group treated with scFv.

### 2.6. The scFv Treatment Reduces Microhemorrhages in the Brains of Mice Infected with PbA

**Figure 6:**
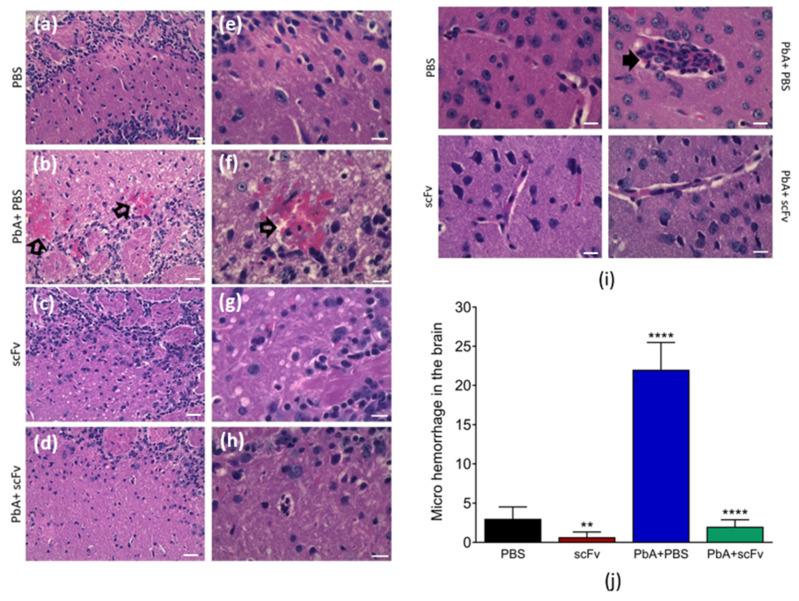
Photomicrographs of brains from mice infected or not with PbA and treated or not with anti-GPI scFv from *Plasmodium*. (**a**,**e**) Representatives of the PBS group (**b**,**f**) Representatives of the PbA+PBS group, microhemorrhages indicated by unfilled arrows. (**c**,**g**) Representatives of the scFv group. (**d**,**h**) Representatives of the PbA+scFv group. (**i**) Leukocyte migration in the brain, evidenced by the filled arrow. (**j**) Quantitative graph of brain microhemorrhages in the mentioned groups. Groups: PBS (*n* = 3), scFv (*n* = 3), PbA+PBS (*n* = 6), PbA+scFv (*n* = 6). The graphs represent mean and standard error of the mean (** *p* < 0.01; **** *p* < 0.0001). Representative images are shown at 20× and 40× (**a**) and 40× (**b**) magnification, H&E staining. The statistical analysis was performed using one-way ANOVA followed by unpaired *t*-test. (**a**–**d**), original magnification: ×20. Scale bar: 20 μm. (**e**–**i**), original magnification: ×40. Scale bars: 40 μm.

**Figure 7:**
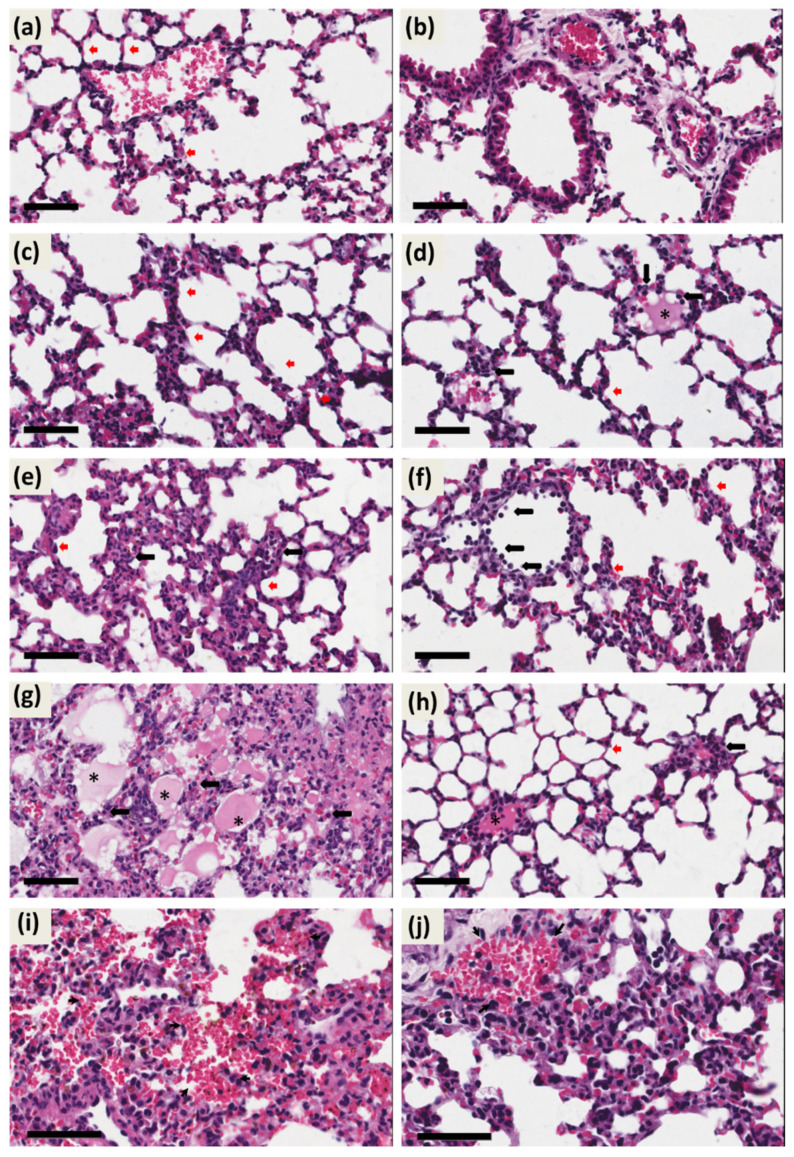
Photomicrographs of the lung demonstrating alterations in the pulmonary parenchyma due PbA. (**a**,**b**) HE-stained lung section showing the pulmonar parenchyma of an uninfected and PBS-treated animal with unchanged lung histology. (**a**–**d**) HE-stained lung section showing the pulmonar parenchyma of an uninfected and scFv-treated animal with mild changed of lung histology such as, slight thickening of the interalveolar septum (red arrow) and infltration (black arrow). (**e**,**g**,**i,j**) Micrographs from the PbA-infected and scFv-untreated group showed thickening of the interalveolar septum (red arrow), infiltrates (black arrow), alveolar edema (asterisks), congestion and hemorrhage (arrowhead). Histopathological images of the PbA-infected and scFv-treated group, it was observed thickening of the interalveolar septum (red arrow), infiltrates (black arrow), isolated areas of edema in the alveolar sac (asterisk) and presence of mild hemorrhage in the parenchyma (arrowhead). Tissue samples were harvested from mice euthanized at day 6 post-infection. (**a**–**i**), original magnification: ×40. Scale bars: 50 μm.

One of the most striking features of severe malaria, which is shared by the ECM model and Falciparum malaria, is cerebral injury accompanied by leukocyte migration, blood leakage into brain tissue with regions of microhemorrhages, and the formation of rosettes [24]. Cerebral tissue lesions are typically associated with the progression of cognitive and motor decline; therefore, we examined the brain tissue of infected animals and scFv-treated. Micrographs of brain tissue on the sixth dpi showed that the group uninfected with PbA and used as control, PBS (Figure 6a) and scFv (Figure 6c) did not present alterations in brain tissue. On the other hand, the group infected with PbA and not treated with scFv (Figure 6b) showed areas of microhemorrhages (open arrows). In images with magnification, the routine staining revealed the presence of microhemorrhages (open arrow, Figure 6b). In this group, leukocyte migration into the blood vessel and subsequent vessel thickening (filled arrow, Figure 6f) were also notable. In contrast, no histological alterations were detected in the brains of the non-infected PBS (Figure 6e) and scFv (Figure 6g) groups. Interestingly, the histopathological results showed that animals infected with PbA and treated with scFv presented fewer regions with microhemorrhages (Figure 6d and Figure 7h). Analysis of brain microhemorrhages revealed a significant statistical difference (*p* < 0.0001) between groups treated and untreated with scFv, demonstrating that scFv provides brain protection to PbA-infected animals as shown in the bar chart on Figure 6j. Microhemorrhages are illustrated in Figure 6f, indicated by unfilled arrows. The number of microhemorrhages was approximately five times higher in the PbA-infected and untreated animals compared to the infected animals that received scFv treatment. Moreover, in scFv-treated and infected animals, no leukocyte migration was detected in the vessels, resulting in better preservation of the blood vessels (Figure 6i).

### 2.7. The scFv Treatment Ameliorates Alterations in Lung Tissue in PbA-Infected Mice

Severe malaria is accompanied by multisystemic complications. In addition to nervous tissue involvement, respiratory failure, liver disorders (hepatomegaly), splenomegaly, and acute kidney injury (AKI) are also observed [25,26,27]. To investigate these complications in animals with ECM, histopathological analysis of the lungs was performed (Figure 7). On 6 dpi, the control animals were also euthanized, and after H&E staining, it was observed that scFv-treated and non-infected animals presented slight thickening of the interalveolar septum (red arrow) and some areas with infiltrates (black arrow; Figure 7c,d, respectively). Isolated and scattered areas of edema were also noted in alveolar sacs (asterisks, Figure 7d), compared to images of PBS-treated and non-infected animals (Figure 7a,b). Representative micrographs from the PbA-infected and scFv-untreated group revealed greater thickening of the interalveolar septum (red arrow) with abundant infiltrates (black arrow) dispersed throughout the lung parenchyma (Figure 7e,g) and more extensive areas with alveolar edema (asterisks; Figure 7g). Additionally, the presence of congestion and hemorrhage was noted (arrowhead; Figure 7g), although this alteration was not widespread throughout the parenchyma (Supplementary Figure S1). On the other hand, when we analyzed representative histopathological images of the PbA-infected and scFv-treated group, it was also observed thickening of the interalveolar septum (red arrow; Figure 7f), but this was milder compared to the untreated and PbA-infected group (Figure 7f), and infiltrates were present (black arrow). In Figure 7h, a portion of the parenchyma shows a delicate alveolar septum, isolated areas of edema in the alveolar sac (asterisk), and infiltrates (black arrow). A smaller area of interalveolar septal thickening in animals that received treatment and PbA-infected was also observed in lower magnification micrographs (Supplementary Figure S1d), compared to PbA-infected and scFv-untreated animals (Supplementary Figure S1c). Another alteration observed was the presence of mild hemorrhage in the parenchyma of scFv-treated and PbA-infected animals (arrowhead, Figure 7j). Despite the histological alterations detected during *Plasmodium* infection and scFv treatment, quantitative results of the alveolar septal area showed no statistically significant differences between the groups studied in this research (Supplementary Figure S2).

### 2.8. The scFv Treatment Protects Hepatic Tissue Against PbA Damage

**Figure 8:**
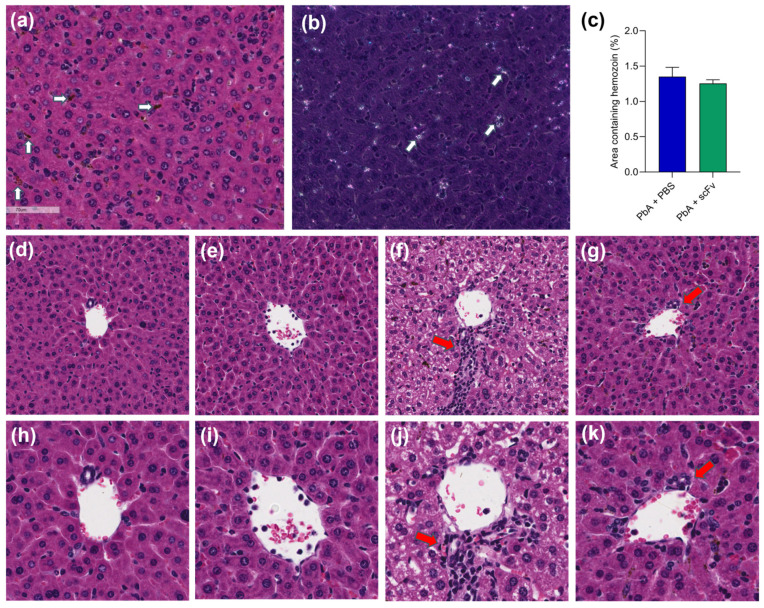
Hepatic injury due PbA is ameliorated with scFV treatment. (**a**) Photomicrographs of the liver showing the presence of hemozoin (white arrows) in HE staining. (**b**) Photomicrograph of the liver under polarized light microscopy, showing the presence of birefringent structures (white arrows) that indicate hemozoin aggregates. (**c**) Graph representing the percentage of area occupied by hemozoin in the groups infected with PbA. PbA+PBS (*n* = 6), PbA+scFv (*n* = 6). In (**a**) the white arrows indicate leukocyte infiltrate. (**d**,**h**) These are representative images of the PBS group, at 20× and 40× magnification, respectively. (**e**,**i**) These are representative images of the scFv group, at 20× and 40× magnification, respectively. (**f**,**j**) These are representative images of the PbA+PBS group, at 20× and 40× magnification, respectively. (**g**,**k**) These are representative images of the PbA+scFv group, at 20× and 40× magnification, respectively. The red arrows in the images represent leukocyte aggregates, black arrows point to hepatic vacuoles and black asterisks show sinusoid capillaries. The graphs show mean and standard error of the mean. Representative images are displayed at 20× magnification, H&E staining. The statistical analysis was performed using the Kolmogorov–Smirnov test followed by unpaired *t*-test with Welch’s correction.

In the ECM model caused by PbA, much has been studied to understand the pathogenesis and immune response in cerebral malaria, respiratory disorders, and liver damage. However, little is known about the role of the parasite’s GPI anchors in the histopathology of these organs. In this study, we also investigated the effects of scFV as an anti-GPI target of the parasite on the hepatic parenchyma. One of the changes in the hepatic parenchyma was assessed through the detection and quantification of hemozoin. Histological results show the presence of hemozoin deposited in the hepatic parenchyma of the group infected with PbA and untreated (white arrow; Figure 8a,b). Morphometric analysis using polarized light microscopy to detect and quantify hemozoin abundance revealed no difference between the untreated and scFV-treated groups (Figure 8c), both infected with PbA at 6 dpi. As expected, hemozoin deposition was not detected in uninfected animals (Figure 8d,e,h,i). Furthermore, histological changes such as inflammatory infiltrates (red arrows) and significant sinusoidal capillary dilation (asterisks) can be observed in the low-magnification image (Figure 8f) of the hepatic parenchyma in PbA-infected and untreated animals at 6 dpi. In addition, in high-magnification images of hepatic tissue, cytoplasmic vacuolization of hepatocytes (black arrows) is visualized in PbA-infected and untreated animals (Figure 8j), compared to control groups. Conversely, in PbA-infected and scFV-treated animals, aside from the presence of hemozoin, only a small amount of infiltrate is observed (red arrow), and no apparent histological tissue alterations in the hepatic tissue were detected (Figure 8g,k).

**Figure 9:**
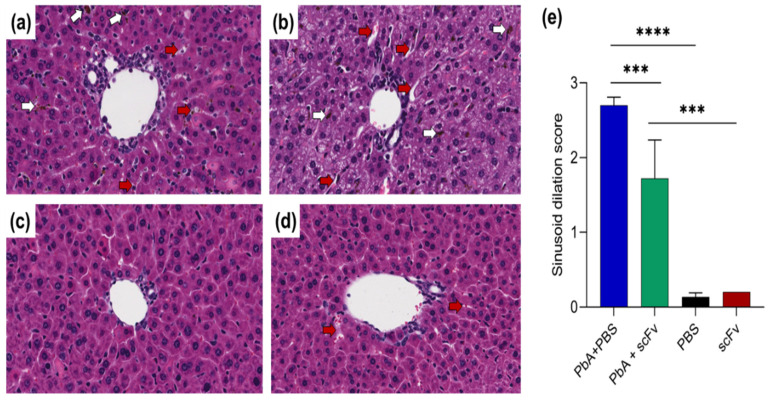
Dilation of sinusoids in the liver of mice infected with PbA is reduced by treatment with scFv. (**a**) Photomicrograph of the liver of animals in the PbA+scFv group (*n* = 6) with red arrows pointing to areas of moderate sinusoid dilation. (**b**) Photomicrograph of the liver of an animal in the PbA+PBS group (*n* = 6) with red arrows showing areas of severe sinusoid dilatation. (**c**) Liver of a healthy animal treated with PBS (*n* = 3). (**d**) Liver of healthy animal treated with scFv (*n* = 3), showing areas of mild sinusoid dilatation. (**e**) Graph illustrating sinusoid dilatation score, where ten liver fields from each animal were analyzed and scored from 0 to 3, where 0—no dilatation, 1—mild dilatation, 2—moderate dilatation and 3—severe dilatation. White arrows indicate accumulation of hemozoin. The graph represents mean and standard deviation. Representative images are displayed at 30× magnification, H&E staining. Statistical analysis was performed using the Kruskal–Wallis test followed by Tukey’s multiple comparisons test. (*** *p* < 0.001; **** *p* < 0.0001).

As for the dilation of the hepatic sinusoids, we quantified them using scores to see if there was a statistical difference between the groups. We found a significant reduction in the dilation of the sinusoids in the group infected with PbA and treated with scFv compared to the infected group treated with PBS (Figure 9e). This indicates that the treatment may be associated with improvements in liver hemodynamics and possibly a reduction in lesions and complications related to blood circulation [28,29].

Histological examination revealed severe sinusoid dilatation in PbA-infected, PBS-treated animals (Figure 9b), while PbA-infected animals treated with scFv showed only moderate dilatation (Figure 9a). Control animals treated with PBS (Figure 9c) or scFv (Figure 9d) showed minimal to mild sinusoid dilatation. Morphometric quantification using a scoring system confirmed a significant reduction in sinusoid dilation in the PbA-infected group treated with scFv compared to the infected group treated with PBS (Figure 9e). This indicates that the treatment may be associated with improvements in liver hemodynamics and possibly a reduction in lesions and complications related to blood circulation.

### 2.9. Treatment with scFv Attenuates Renal Tissue Damage Caused by PbA

**Figure 10:**
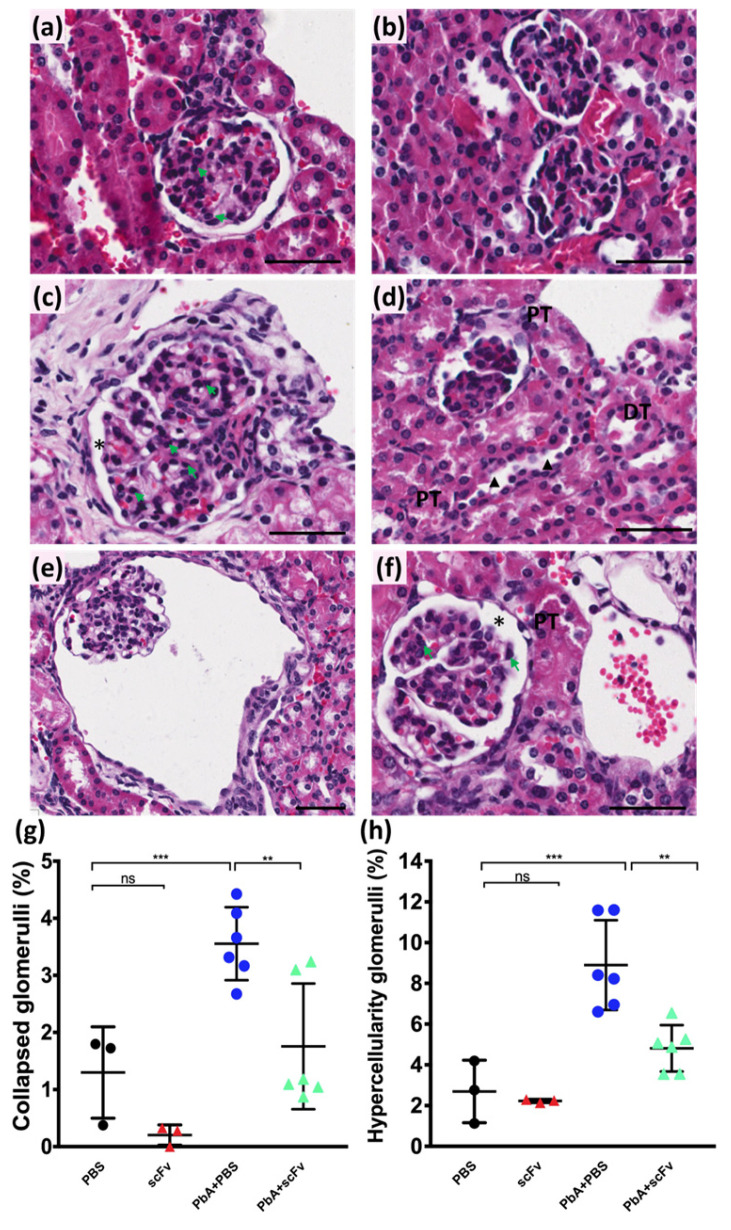
Histological alterations in the renal corpuscle of C57BL/6J mice infected with PbA and treated with scFv. C57BL/6J mice were infected with PbA, and the kidney was collected on the six-day post-infection. Histological sections are representative of control animals, not infected with PbA; PBS-treated (**a**) and scFv-treated (**b**). Sections (**c**–**f**) are representative of C57BL/6J mice infected with PbA. (**c**,**f**) The histological section shows a renal corpuscle of PbA-infected animals with an increased number of mesangial cells in the glomerulus (green arrow) compared to non-infected animals (**a**,**b**). (**d**,**e**)—Histological sections of PbA-infected animals show the presence of collapsed glomeruli and loss of brush border (black arrow heads) in proximal tubule (PT); distal tubule is shown as well (DT). (**f**) The PbA-infected and scFv-treated group presented a mild increase in Bowman’s capsule space (asterisk), with glomeruli showing fewer mesangial cells (green arrow). (**g**) Percentage of glomeruli with collapsed tufts in the renal parenchyma of PbA-infected and PBS-treated (*n* = 6) and PbA-infected and scFv -treated animals (*n* = 6), showing 3.5% and 1.7% of collapsed glomerulli, respectively (** *p* = 0.009). No difference was detected to control PBS-treated (*n* = 3) and scFv-treated (=3). (**h**) percentage of mesangial cells detected in the whole section renal parenchyma of the organ from non-infected animals treated with PBS (*n* = 3), non-infected animals scFv-treated (*n* = 3), PbA-infected animals (*n* = 6), and PbA-infected animals scFv-treated (*n* = 6). Circles represent the animals in both PBS groups, whilst triangles indicate the animals in both scFv groups. The black circles indicate the individual animals comprising the PBS group, whilst the blue ones indicate those in the PbA+PBS group. The red triangles, meanwhile, indicate the individual animals comprising the scFv group, whilst the green ones indicate those in the PbA+scFv group. The statistical analysis of morphometric results showed a difference between the PbA-infected and PBS-treated (*n* = 6) and PbA-infected and scFv-treated (*n* = 6) groups, revealing 8.8% and 4.8% of hypercellularity glomeruli, respectively (** *p* = 0.002). Conversely, the controls showed no differences between them. Tissue samples were harvested from mice euthanized at day 6 post-infection and HE staining was performed. (**a**–**f**), original magnification: ×40. Scale bars: 40 μm. *** *p* < 0.001; ‘ns’ indicates not significant.

As previously mentioned, severe cases of Falciparum malaria can progress to acute kidney injury (AKI). Similarly, in animal models, AKI associated with ECM has been identified; therefore, on the sixth dpi with PbA, we investigated the histopathological alterations associated with AKI in this model, along with the effects of scFv treatment [30,31,32,33]. Histological images of HE-stained whole kidney parenchyma sections from PbA-infected and PBS-treated animals revealed an increase in Bowman’s capsule space (asterisk) and hypercellularity (green arrow; Figure 10c), and a greater number of collapsed glomeruli (tufts, Figure 10d,e), while these alterations were not observed in the controls, uninfected PBS-treated (Figure 10a) and uninfected scFv-treated group (Figure 10b). Conversely, the PbA-infected and scFv-treated group (Figure 10f) exhibited a mild increase in Bowman’s capsule space (asterisk), with glomeruli showing fewer mesangial cells (green arrow), characteristic of reduced hypercellularity. To quantify the number of collapsed glomeruli and hypercellularity, a morphometric analysis was conducted on whole kidney sections. On day 6 post-infection, a statistically significant increase of approximately 3.6% in collapsed glomeruli was observed in the PbA-infected and PBS-treated group (*n* = 6), while the PBS- and scFv-treated control groups showed 1.3% and 1.0%, respectively (*n* = 3 for both treatments, Figure 10g). Furthermore, there was no significant difference in the percentage of collapsed glomeruli between the PbA-infected and scFv-treated group (1.7%) and the controls (Figure 10g). Morphometric analysis of glomerular hypercellularity revealed a significant increase of approximately four-fold (8.8%) in the PbA-infected and PBS-treated group (*n* = 6) compared to the PBS- and scFv-treated controls (2.2%, *** *p* = 0.0004 and 2.1%, ** *p* = 0.0002, respectively; Figure 10h). Interestingly, the results indicated that the PbA-infected and scFv-treated group (*n* = 6) tended to exhibit greater glomerular cellularity than the controls (*n* = 3 for both); however, these differences were not statistically significant (Figure 10h). Furthermore, the PbA-infected and scFv-treated group (4.1%; ** *p* = 0.0027) exhibited approximately 50% less glomerular cellularity than the untreated PbA-infected group (8.8%). One of the alterations observed in AKI associated with malaria in other studies is tubular damage [30,31,32,33]. On the sixth day post-infection, the PbA-infected and PBS-treated group exhibited a loss of brush border in a few proximal convoluted tubules (black arrowhead, Figure 10d); however, this alteration was not detected in renal parenchyma of all animals in this group. Additionally, only minor tubular alterations were observed in the PbA-infected and scFv-treated group at day 6 dpi. This set of results reveals that scFv treatment may mitigate renal tissue damage caused by cerebral malaria complications.

## 3. Discussion

Cerebral malaria is a serious neuropathological complication caused by *Plasmodium* spp. Its clinical manifestations are characterized by severe anemia, coma and convulsions, with the possibility of progressing to death [34]. In addition to the incomplete elucidation of its pathogenesis, *Plasmodium* has shown resistance to current treatments, representing a significant global concern [35,36].

By using animal models, we can investigate new drugs and the mechanisms involved in the disease [37]. C57BL/6 mice infected with PbA constitute a well-established model that shares many similarities with human cerebral malaria, including the sequestration of infected red blood cells in the brain, neurological signs such as paralysis, ataxia, seizures and respiratory distress [38,39,40].

Single-chain variable fragments (scFv) are structures with similar functions to antibodies, but with some advantages: they have a greater ability to penetrate tissues and reach them more quickly and uniformly due to their smaller size [41,42]. Several scFv have been selected for specific therapies, such as cancer treatment and antiviral applications, but little is known specifically about the GPI of *P. falciparum* [43,44].

In this study, we sought to understand the effect of a *Plasmodium* anti-GPI scFv selected by Phage display on murine cerebral malaria. Initially, we performed a survival curve, rescuing 71.4% of the animals, which is highly promising, considering that mice infected with PbA usually succumb between the 6th and 10th day post-infection, exhibiting neurological signs in the last few days or hours [45].

In addition, when monitoring body weight, all groups showed initial weight loss, probably due to factors related to stress and acute inflammation caused by the administration of the parasite [45]. The progressive decrease in weight in the PbA+PBS group from the third day post-infection (dpi) can be attributed to parasitemia and reduced food and water intake [46]. On the other hand, the infected animals treated with scFv were active and showed an increase in body weight on days 2, 3, 5 and 6 post-infection, indicating a general improvement in the disease condition. As mice lose weight rapidly in the presence of infectious diseases, this assessment can be used to predict the possibility of the animal’s death, aligning with our survival curve and body weight results [47]. Thus, we observed that the weight recovery induced by treatment with scFv indicates an improvement in the general condition of the disease and greater chances of survival.

These findings align with the indications of morbidity demonstrated in the RMCBS scale (Figure 3), where statistically higher scores were observed in animals treated with scFv. It is noteworthy that higher scores correspond to fewer clinical signs, indicating that scFv protected animals from severe cerebral malaria symptoms. According to Carroll et al. [48], RMCBS scores of 10 or less characterize signs of cerebral malaria, and scores of 5 or less result in animal death between 1 and 4 h, thus requiring euthanasia.

The score assessments can be divided into two major groups. The first includes body position, grooming, ocular assessment, and motor performance. These parameters characterize general disease signs in mice, as healthy animals dedicate the majority of their time to activities such as grooming (approximately 40%), followed by exploration, locomotion, and feeding [47]. Thus, we understand that symptoms such as hunched posture, piloerection, reduced self-cleaning, eye closure and secretion, as well as decreased exploratory activity, observed in both infected groups and more pronounced in the PbA+PBS group, are expected and represent an organism’s mechanism to conserve energy in disease situations [47]. Despite the manifestation of these signs, we observed higher scores in animals from the PbA+scFv group, indicating that the treatment resulted in an improvement in the overall clinical condition.

The second major group is directed towards the evaluation of the central and peripheral nervous systems, including gait, balance, strength of thoracic limbs, response to touch, paw reflex, and pinna reflex. Animals infected and treated with scFv showed no alterations in these parameters, unlike those infected and treated with PBS, which exhibited signs ranging from moderate to severe, especially on the 6th dpi. This suggests the preservation of the integrity of the blood–brain barrier and the central nervous system concerning inflammation, edema formation, hemorrhages, and hypoxia in the scFv-treated group. These events are known to contribute to increased intracranial pressure and microlesions in brain tissue, resulting in neurological signs associated with cerebral malaria [7,25,49].

Acute cerebral malaria is associated with the adherence of infected erythrocytes to the vascular endothelium, and when associated with high parasitemia, exacerbates the formation of microthrombi, microhemorrhages, and edema in various organs, especially the brain [39]. Thus, the blood parasitic load holds significant importance in the pathogenesis of cerebral malaria, as the rupture of the blood–brain barrier leads to the sudden formation of cerebral edema, causing severe neurological signs and potential fatality [7,39]. With our treatment, we observed a reduction in parasitemia, particularly on days 5 and 6 post-infection, along with a significant decrease in cerebral hemorrhages. This suggests a direct correlation with improvements in morbidity and mortality indicators [50,51,52].

In hematological analyses, comparing the PbA+scFv group with the PbA+PBS group, a significant reduction in monocytosis, an increase in platelet count, and a decrease in MCHC were observed. The results of the infected group treated with PBS were similar to the findings of Kotepui et al. [53], who analyzed hematological parameters in 4985 human patients, including 703 with malaria. Both the mice in this study and the human patients in Kotepui et al.’s study showed low platelet, leukocyte, and lymphocyte counts, along with increased monocyte and neutrophil counts. Additional studies in humans have also reported similar alterations, such as thrombocytopenia, leukopenia, lymphocytopenia, and monocytosis [53]. This indicates that cerebral malaria has an exacerbated systemic inflammatory response, involving pro-inflammatory cytokines, activation of endothelial cells, sequestration of parasitized erythrocytes, platelets and leukocytes, resulting in increased vascular permeability, edema and hemorrhages [25,39].

Cerebral malaria exhibits a significant Th1 response, characterized by an increase in the production of cytokines IL-12p40 and IFNγ. The elevated levels of these cytokines in animals infected and treated with scFv, compared to those treated with PBS, may have reflected an early and effective immune response regulated by pro-inflammatory Th1 cytokines. Particularly, IFN-γ may have played a role in limiting the progression from uncomplicated to complicated malaria, as indicated by the morbidity scores described above [54,55,56].

Th1 cell responses and IFNγ production have also been associated with host resistance during the *Plasmodium* infection hepatic stage. Experimental evidence suggests that liver-resident T cells play a crucial role in eliminating *Plasmodium*-infected hepatocytes through phagocytosis by macrophages, thus preventing the emergence of merozoites and the subsequent manifestation of clinical malaria [57]. The phagocytosis of *Plasmodium*-infected cells, in turn, leads to hemozoin deposition in the liver, which can further induce the pro-inflammatory response [58,59,60]. In our treatment, we observed no changes in hepatic hemozoin deposition, despite lower blood parasitemia, increased hepatic levels of IFNγ and IL-12p40 and a decrease in monocytes in the circulation, which may be associated with the migration of macrophages to the liver.

Despite the more pronounced hepatic pro-inflammatory response in the animals treated with scFv, the presence of less dilated sinusoid capillaries was observed in these animals. This finding is probably due to less congestion and blood stasis as the parasite load decreases [61]. With fewer parasites, there is a reduction in microcirculation obstruction due to the adhesion of parasitized red blood cells and inflammatory cells to the sinusoidal endothelium [62].

In the lungs, there was an increase in IL-12p40 and a decrease in IL-10 in the PbA+scFv group and a decrease in IL-12p40 and an increase in IL-10 in the PbA+PBS group (Figure 5), which demonstrates a suppression in the anti- inflammatory response and increased inflammatory response caused by scFv. Despite there being no difference in the thickening of the alveolar septum (Figure 7), these data are characteristic of an increase in inflammatory cells, in line with the findings of Aitken and collaborators, who observed a marked leukocyte infiltrate without fibrosis, since cerebral malaria presents as an acute condition [63,64].

In the brain, there was no difference in the profile of pro-inflammatory cytokines, but a significant reduction in the presence of microhemorrhages was observed in the histopathology of the PbA+scFv group compared to the PbA+PBS group, suggesting that the protective mechanism observed is independent of classic systemic inflammation pathways mediated by IFNγ and IL-12p40. The absence of a difference in the levels of pro-inflammatory cytokines, combined with the reduction in hemorrhages, suggests that anti-GPI scFv may act by stabilizing the BBB via modulation of adhesion molecules such as ICAM-1 and VCAM-1 and protecting the endothelium from excessive activation induced by GPI [64,65]. These findings corroborate previous studies [19,39], in which the pathology of cerebral malaria is more associated with mechanical and inflammatory disruption of the BBB than with direct parasite infiltration [39,66]. Therefore, scFv seems to exert protection specifically at the endothelium–blood interface, preventing vascular extravasation without largely modulating the cerebral immune response at the most critical moment of the infection.

Acute kidney injury is a known complication in cases of cerebral malaria, associated with endothelial dysfunction, parasite sequestration, and immune-mediated tissue damage [67,68]. Undoubtedly, elevated levels of systemic proinflammatory cytokines—such as IFN-γ, IL-6, IL-1β, and TNF-α—contribute to pathological alterations in both brain and kidney tissues. Our group has previously demonstrated that renal alterations in the ECM model are accompanied by glomerular and tubular damage by day 7 post-PbA infection [30]. In the present study, we also confirmed the presence of tubular changes and glomerular alterations in renal tissue as early as day 6 post-infection. However, animals infected with PbA and treated with anti-GPI antibodies derived from *P. falciparum* exhibited attenuated renal histopathological alterations. Our results suggest that the GPI anchor from *P. falciparum* may reduce parasitemia and, consequently, attenuate the immune response, leading to a lower concentration of circulating parasitic and host molecules and resulting in reduced glomerular and tubular injury. Indeed, GPI anchors synthesized during the trophozoite stage of the intraerythrocytic cycle are crucial for anchoring the MSP-1 protein, which facilitates the invasion process of newly formed merozoites [68]. Furthermore, it is well established that *Plasmodium* GPI anchors activate Toll-like receptors (TLRs), inducing the production and secretion of proinflammatory cytokines such as TNF, as well as the expression of cell adhesion molecules [69]. Within this context, we propose that the scFv may prevent renal injury not solely by reducing parasitemia, but also by decreasing the number of monocytes, which, when activated, produce TNF and IL-1β during *Plasmodium* infection.

It is important to note that while our study demonstrates significant protective effects in the brain and kidneys, the lack of statistically significant improvements in lung alveolar septal thickening and hepatic hemozoin deposition suggests that the anti-GPI scFv mechanism may be organ-specific or that different pathological processes respond differently to this intervention. Alternatively, the 6-day timepoint may be too early to detect significant changes in hemozoin clearance, or higher doses/different dosing schedules may be required for pulmonary and hepatic protection. Future studies should include multiple timepoints, dose–response analyses, and additional quantitative parameters to fully characterize the therapeutic potential of anti-GPI scFv across all affected organs.

Therefore, scFv treatment yielded numerous positive findings in malaria pathogenesis, preventing the disease from progressing to cerebral complications. Further studies are necessary for a comprehensive understanding of the involved mechanisms, but we believe that an initial step has been taken toward a potential new treatment.

## 4. Materials and Methods

### 4.1. Ethics Statement

All studies involving mice were previously approved by the Ethics Committee on Animal Use at the Federal University of Uberlândia (CEUA/UFU), under protocol number 153/16. All animal handling and welfare procedures were conducted in accordance with the recommendations of the National Council for Animal Experimentation Control (CONCEA) through its normative resolutions and technical guidelines [23]. The animals were housed in the Central Animal Facility of the Rodent Bioterium of the Federal University of Uberlândia (REBIR-UFU), which is registered with CONCEA (CIAEP: 01.0105.2014) and the National Biosafety Technical Commission—CTNBio (CQB: 163/02).

In addition, the team that conducted the in vivo experiments took training courses in animal handling and experimentation through the University of São Paulo, the Oswaldo Cruz Foundation, or the Brazilian Society for Animal Experimentation (depending on each researcher).

### 4.2. Expression and Purification of scFv

The selection of GPI-binding scFvs was carried out using the Phage display technique, where an analysis of the specificity of the selected targets and their subsequent sequencing was carried out. With the isolated clones, scFv expression was increased and the supernatant containing scFv was subsequently purified using surface layer chromatography with the HisTrapTM HP column (GE Healthcare Life Sciences, Waukesha, WI, USA), following the manufacturer’s instructions, on an HPLC system (AKTATM purifier, GE Healthcare Life Sciences, Waukesha, WI, USA).

After nickel immobilization on the column and the addition of binding buffer (50 mM Imidazole (I5513, Sigma-Aldrich, St. Louis, MO, USA), 0.5 M NaCl (7647-14-5, Sigma-Aldrich, St. Louis, MO, USA), 20 mM Na2HPO4 (71505, Sigma-Aldrich, St. Louis, MO, USA), pH 7.4), the supernatant from the bacterial culture, previously obtained, was added. The scFv molecules, bound to nickel by the hexa-histidine (His6) tail, were eluted through competition with elution buffer (500 mM Imidazole, 0.5 M NaCl, 20 mM Na2HPO4, pH 7.4). The purified scFv was concentrated using a 3 kDa Amicon tube (Millipore, Billerica, MA, USA), and the protein concentration was determined by gel band quantification through electrophoresis and analysis using the ImageJ program (version 1.50i). The treatment dose was determined to be 100 μg per animal.

### 4.3. Sample Size Calculation

The sample size was calculated using G*Power software (version 3.1.9.7), using a one-way ANOVA statistical test (fixed effects, comparison between multiple groups). An effect size (f) of 0.82 was considered, based on the magnitude observed in previous studies conducted by our group involving the evaluation of parasite load in *Plasmodium* infection models and treatments with scFv. The statistical power was set at 95% (1 − β = 0.95) and the type I error (α) at 5% (α = 0.05). The effect size was estimated according to Cohen’s definition for F tests. The number of groups (*n* = 4) corresponds to the different treatments and controls described in Section 4.6 of this article. Considering these parameters, the analysis indicated a total sample size of 32 animals, which is sufficient to detect significant differences between groups, even considering possible experimental losses. In addition, six other animals not included in the group calculation were included, which were used to maintain the parasite until experimental infection. One animal was excluded before the start of the experiments due to the presence of anophthalmia; no other animals were excluded during the course of the experiment. Thus, we used 37 animals, 31 of the C57BL/6 wild-type (WT) strain and six of the BALB/c WT strain.

### 4.4. Animal Housing Conditions

All mice used, both C57BL/6 and BALB/c strains, were female, between six and eight weeks old, kept under specific pathogen-free (SPF) conditions, and clinically healthy. The animals were housed in Alesco Ventilife microisolators (Alesco, Monte Mor, SP, Brazil), with autoclaved pine shavings bedding changed weekly, filtered and autoclaved water provided ad libitum, and irradiated Nuvilab CR-1 feed (Quimtia, Colombo, PR, Brazil) also provided ad libitum. Environmental conditions were maintained at a room temperature of 22 ± 2 °C, humidity between 40 and 60%, and a 12 h/12 h light/dark photoperiod cycle. The cages of all animals in this study were placed at the same height on the shelf and kept close to each other and in the same location throughout the experiment. Handling and all in vivo experimental procedures were performed by the same researchers throughout the experiment. Acclimatization occurred from the third or fourth week of life (depending on the age of the animals provided), with three acclimatization episodes per week.

The allocation of animals to experimental groups was not performed blindly due to limited personnel to conduct all in vivo procedures. However, the biological samples collected (such as blood, tissues, and blood smears for parasitemia) were coded before laboratory analysis, ensuring that the evaluators were blinded to the identity of the experimental units during the quantification and data analysis stages.

### 4.5. Parasite Maintenance and Experimental Infection

BALB/c mice were used for maintenance of the PbA parasite due to their greater resistance and subsequent appearance of clinical signs, which are less severe compared to C57BL/6 mice [70]. An aliquot of PbA-infected blood, kindly provided by Dr. Ricardo T. Gazzinelli, was thawed and immediately inoculated into a BALB/c mouse. After 10 days, with daily monitoring of the animal’s clinical condition and parasitemia, a blood sample was collected, and the process was repeated twice as many times, completing three passages before the start of the experimental infection itself. Passages are allowed to stabilize the strain and maintain uniform characteristics of the parasite in all experiments [71]. This thawing and passage process was performed twice, once for the survival experiment and once for the experiment in which we collected biological samples for analysis, so in total, we used 6 BALB/c mice.

On the day of experimental infection, approximately 500 µL of blood was collected from the maintenance animal, which had been previously anesthetized with 3% to 4% isoflurane (#ISO-6, Torrington, CT, USA) and sacrificed by cervical dislocation after collection. A blood smear was performed to determine the percentage of parasitized red blood cells. Subsequently, the blood was diluted in PBS (806544, Sigma-Aldrich, St. Louis, MO, USA), and the number of red blood cells per ml was determined using a Neubauer chamber. This allowed the calculation of the dilution factor necessary to achieve 1 × 10^6^ parasitized red blood cells in each 200 µL of the solution (blood+PBS) to be inoculated into each animal.

### 4.6. Experimental Design

The animals, in this case our experimental units (*n*:18), were randomized by body weight blocks (stratified randomization) into experimental groups as follows:

- PbA+PBS: Animals infected with PbA strain and treated with PBS (6 animals).
- PbA+scFv: Animals infected with PbA strain and treated with the Single-Chain Variable Fragment against GPI of *P. falciparum* (6 animals).
- PBS: Non-infected animals treated with PBS (3 animals).
- scFv: Non-infected animals treated with the Single-Chain Variable Fragment against GPI of *P. falciparum* (3 animals).

**Figure 11:**
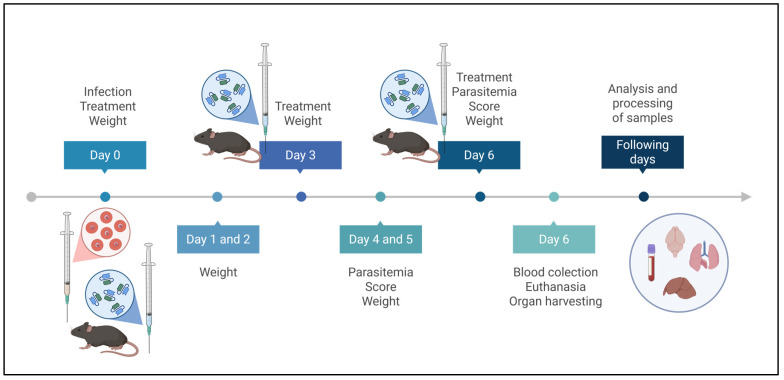
Representative illustration of the experimental design. On day 0 of the experiment, animals were challenged with PbA and received the first dose of treatment; the second and third doses were administered on the 3rd and 6th days post-infection (dpi), respectively. Mice were assessed for body weight daily, parasitemia (4th to 6th dpi), and morbidity score (4th to 6th dpi). On the 6th dpi, animals were anesthetized for blood collection, subsequent euthanasia, and organ harvesting. Original image created in BioRender. Costa polveiro, R. (2026) https://BioRender.com/3nqpe24.

On day 0 of the experiment, the animals received the first treatment dose intraperitoneally. Four hours later, they were challenged with PbA via the same route. The second and third doses were administered on days three and six post-infection (dpi), respectively. The mice were evaluated daily for body weight, parasitemia (days 4–6 post-infection [dpi]), and morbidity index (days 4–6 dpi). All treatments, weightings, evaluations, and parasitemia assessments were performed in the morning. On the 6° dpi, in the afternoon, the animals were anesthetized with 3.5–4% isoflurane for blood collection. While still under anesthesia, they were euthanized by cervical dislocation, followed by organ collection. Blood samples were used for hematological analysis. The collected organs included the brain, liver, and lungs, from which cytokine (IL-10, IL-12p40, and IFN-γ) and histopathological analyses were performed. The experimental design is shown in Figure 11.

The reasons for choosing these days were as follows:

- Day 0—The first dose was administered 4 h before parasite challenge to ensure adequate scFv bioavailability at the onset of infection. This timing allows the antibody fragment to be present during the initial stages of parasite invasion and GPI release, potentially preventing early endothelial activation and the cascade of inflammatory events that characterize ECM pathogenesis.
- Day 3—By day 3 post-infection, the parasite has completed initial replication cycles, and the inflammatory response begins to intensify. GPI anchors are released during schizont rupture, activating Toll-like receptors and inducing production of pro-inflammatory cytokines (TNF-α, IL-1β, IFN-γ). The second dose at this critical timepoint aims to neutralize accumulating GPI molecules and modulate the escalating immune response before severe complications manifest.
- Day 6—Day 6 represents a critical juncture in ECM pathogenesis. Our results (Figure 1a and survival curve) demonstrate that untreated animals begin succumbing to infection between days 7–9, with the first death occurring on day 7. Furthermore, as shown in Figure 2b, parasitemia in untreated animals reaches 10.93 ± 4.05% by day 6, compared to 3.02 ± 0.60% on day 4. This exponential increase means substantially more GPI is being released into circulation on days 5–6, requiring continued antibody neutralization to prevent terminal complications.

### 4.7. Survival Analysis

A separate experiment was conducted to generate the survival curve, with the animal model, scFv processing, and parasite conditions following the same procedures described above. The groups included PbA+PBS (*n*:6) and PbA+scFv (*n*:7), totaling 13 experimental units. The animals were monitored for 30 days after infection with two daily checks (8 a.m. and 3 p.m.) for the following signs to consider the humane endpoint:

- Presence of deep/glazed eyes and/or excessive secretion
- Lack of grooming
- Loss of ≥20% of body weight
- Presence of neurological signs: motor incoordination, spasms, paralysis, and/or reluctance to move
- Prolonged prostration
- Labored breathing
- Persistent body curvature
- Dehydration ≥ 10% (when the skin took more than 2 s to return to normal when checking skin turgor)

Not all signs needed to be present to consider the outcome, but if a set of these signs demonstrated that the animal had a characteristic case of cerebral malaria, the animals were immediately induced into anesthesia with isoflurane (3 to 4% rate) and then sacrificed by cervical dislocation.

Deaths for the survival curve were counted whenever the humane endpoint had to be applied, and the day of death was counted regardless of the time of day it occurred. No animals were found dead during the checks.

### 4.8. Body Weight

To assess whether infection and treatment could impact the animals’ body weight, they were weighed from day 0 to day 6 post-infection (dpi), always in the morning.

### 4.9. Morbidity

Morbidity was assessed using the Rapid and Quantitative Scale of Murine Coma and Behavior (RMCBS), adapted from Carroll [48].

This scale evaluates the clinical picture of cerebral malaria, consisting of 10 parameters, and was developed to assess manifestations of cerebral malaria in C57BL/6 mice infected with PbA The RMCBS allows us to monitor the progression of the clinical syndrome, validated here by correlations with intracerebral hemorrhages and edema that can cause different neurological alterations, depending on the affected brain region. It provides a tool by which animals can be identified as symptomatic or asymptomatic, as well as evaluated for the progression and/or regression of the condition during experimental treatment.

Each animal was evaluated for an average of 3 min. This assessment was conducted once a day on the 4th and 5th dpi and twice on the 6th dpi, once in the morning and once in the afternoon. The total score consists of 10 parameters, each of which is assigned a score ranging from 0 to 2, with 0 related to more severe symptoms, 1 to intermediate, and 2 to low or absent signs of morbidity. Thus, animals with higher scores demonstrate better clinical condition. These parameters were compiled as a sum of scores, as well as evaluated separately.

### 4.10. Parasitemia

Each mouse had a drop of blood collected by puncture of the lateral tail vein using a disposable 26G needle (13 × 0.45), which was used to prepare thin blood smears on microscopy slides (Precision Glass Line) on days 4, 5, and 6 post-infection. The slides were then stained with Rapid Panoptic (Laborclin, Pinhais, PR, Brazil) and subsequently visualized under an optical microscope (Olympus BX40, Olympus, Tokyo, Japan). Five to six representative fields of the slide, each containing approximately 200 erythrocytes, were photographed to allow the counting of approximately 1000 erythrocytes per animal per day of infection. The counting was performed using the ImageJ software (version 1.50i), where infected erythrocytes and total erythrocytes from these fields were counted to calculate the percentage of parasitemia.

### 4.11. Hematology

Blood for hematological analyses was collected via retro-orbital route using a glass capillary and a microtube with a capacity of 500 μL containing EDTA K2 anticoagulant (ethylenediaminetetraacetic acid dipotassium). The collected volume was approximately 100 μL per animal. Hematological analyses were performed using the BC-2800 (Mindray, São Paulo, SP, Brazil) vet equipment, where the following parameters were analyzed: red blood cell count (RBC), hemoglobin (HGB), hematocrit (HCT), mean corpuscular volume (MCV), mean corpuscular hemoglobin (MCH), mean corpuscular hemoglobin concentration (MCHC), red cell distribution width (RDW), in addition to the total white blood cell (WBC) analysis. With the total white blood cell data obtained from the equipment, a white blood cell differential count was performed through blood smears under optical microscopy.

### 4.12. Cytokines

The determination of cytokines was performed through Enzyme-Linked Immunosorbent Assay (ELISA). The concentrations of IFN-γ, IL-10, and IL-12p40 were measured in homogenates of brain, lung, spleen, and liver (100 mg of tissue) from each mouse. The quantification of cytokines was carried out using commercial ELISA kits, following the manufacturer’s recommended protocols (BD Biosciences, San Diego, CA, USA). High-affinity 96-well plates (Corning Laboratories Inc., New York, NY, USA) were coated with specific anti-mouse capture antibodies for IFN-γ, IL-10, and IL-12p40 and incubated overnight at 4 °C. Subsequently, to block nonspecific sites, 10% fetal bovine serum in 0.01 M PBS (pH 7.2) was added to the plate for 1 h. After blocking, standard curves of the respective cytokines in serial dilutions and test samples were added to the plates and incubated for 2 h. For quantifying the presence of cytokines in the samples, biotin-conjugated anti-mouse cytokine detection antibodies, supplemented with streptavidin-peroxidase, were incubated for 1 h. Between each step, plates were washed with PBS-T 0.05% (PBS-T). The reaction was developed with tetramethylbenzidine (TMB), followed by the addition of 2M sulfuric acid to stop the reaction. Optical density (OD) was determined at 450 nm using a microplate reader (SpectraMax M2e, Molecular Devices, San José, CA, USA). Cytokine concentrations were determined from the standard curve with known cytokine concentrations, and the results were expressed in pg/mL, according to the detection limits for each assay: IFN-γ (4.1 pg/mL), IL-10 (4.5 pg/mL), and IL-12p40 (15.6 pg/mL).

### 4.13. Histology

Brain, lung, liver, and kidney samples were collected on the 6th day post-infection (dpi) immediately after euthanasia. The organs were fixed in 10% buffered formalin at room temperature for 24 h, and then formalin was replaced with 70% alcohol until the paraffin embedding process. After embedding, the paraffinized organs were sectioned (5 μm thick) and placed on microscopic slides, followed by deparaffinization and hydration for Hematoxylin (H3136, Sigma-Aldrich, St. Louis, MO, USA) and Eosin (E4009, Sigma-Aldrich, St. Louis, MO, USA) (H&E) staining. The slides were digitized using the Scanscope AT scanner (Leica Microsystems GmbH, Wetzlar, Germany).

### 4.14. Alveolar Septum Measurement

From the digitized histological slides, lung images were acquired at a 20× magnification and divided into 10 microscopic fields using the QuPath software (version 0.7.0). These fields were then analyzed for the thickness of the pulmonary septal area.

The septal thickness analysis was performed using ImageJ software. The quadrants separated in the previous process had their images converted to 8-bit quality. A threshold was applied, and subsequently, the total percentage of the “dark area,” indicating the septal region, was calculated in comparison to the overall region.

### 4.15. Hemozoin Quantification

Quantitative analysis of hepatic hemozoin (Hz) was conducted by reading H&E-stained histological sections observed under polarized light microscopy. Image captures were performed using a 20× objective on the Nikon Eclipse Ti-5 microscope (Nikon, Tokyo, Japan). Ten microscopic fields from each tissue section of each animal were analyzed using a polarized filter to identify the disorientation caused by Hz. Images were captured using the adapted polarized light filter and images using conventional light microscopy. Using ImageJ software, version 1.50i, each image was converted to 8 bits, transforming colored images into grayscale. The threshold was adjusted to highlight pixels covered by hemozoin in red. These areas were measured and expressed as a percentage of the total pixel area.

### 4.16. Sinusoidal Dilatation Score

One of the histological characteristics employed to evaluate the severity of liver lesions is the dilatation of the sinusoids. To that end, the sinusoidal dilatation was graded using scores based on the work by Techarang [72]: (0) No dilatation, (1) Mild dilatation, (2) Moderate dilatation, and (3) Severe dilatation. Ten fields of portal vein regions from each animal were evaluated at 40× magnification, and the average for each individual and then for each group was taken.

### 4.17. Brain Microhemorrhages

A quantitative analysis was performed to assess the number of microhemorrhage foci, and the results were correlated with the RMCBS scores assigned to each animal included in the study. Midsagittal brain sections from infected mice were collected on day 6 post-PbA infection and stained with hematoxylin and eosin (H&E) [30]. The stained sections were scanned using the Aperio ScanScope AT Turbo system (Leica, Heerbrugg, Switzerland), and the resulting images—representing the entire midsagittal section (MSS) of each brain—were examined for hemorrhagic foci using the Aperio ImageScope software version 8.1 (Leica, Heerbrugg, Switzerland).

### 4.18. Morphometric Analysis of Renal Parenchyma

Digital photographs were taken from the kidney slides with a 40× objective. A microscope equipped with a DFC450 camera system (Leica, Wetzlar, Germany) was used to capture the images. Morphometric analysis of hypercellularity was performed using ImageJ v.1.50i image processing software, according to the protocol described and established by Yashima [73]. Glomerular cellularity was measured as the percentage of glomeruli with more than 11 mesangial cells per section. A minimum of 50 glomeruli were analyzed for each animal.

### 4.19. Statistical Analysis

Statistical methods were employed to analyze various parameters in this study. For hemogram data, a one-way ANOVA followed by Tukey’s multiple comparison test was utilized to determine significant differences between groups. Body weight differences were assessed using Student’s *t*-test. Parasitemia levels were analyzed using two-way ANOVA, followed by Sidak’s and Bonferroni’s multiple comparison tests to identify variations between experimental conditions. Morbidity scores were evaluated using two-way ANOVA, followed by Tukey’s multiple comparison test. Survival analysis was conducted using the Kaplan–Meier method, with the Log-rank test (Mantel–Cox) employed to compare survival curves between groups. Cytokine levels were subjected to two-way ANOVA, followed by Holm–Sidak’s and Tukey’s multiple comparison tests to identify significant differences for hemozoin quantification, the Kolmogorov–Smirnov test was initially applied to assess data distribution, followed by the unpaired *t*-test with Welch’s correction to compare means between groups. Microhemorrhages in the brain were analyzed using one-way ANOVA, followed by the unpaired *t*-test. Furthermore, the area of septal tissue in the lungs was evaluated using the non-parametric Kruskal–Wallis test, followed by Dunns’ multiple comparison tests to determine significant differences between experimental groups. The analyses were performed using GraphPad Prism Software, version 9.0, and data were considered statistically significant when *p* < 0.05.

## 5. Conclusions

With our findings, we were able to evaluate the effect of an anti-GPI scFv from *P. falciparum* on cerebral malaria in a mouse model. Anti-GPI scFv treatment significantly improved survival, reduced parasitemia, ameliorated neurological signs, and decreased brain microhemorrhages (*p* < 0.0001) and renal glomerular hypercellularity (*p* = 0.0027). Hepatic sinusoid dilation was also significantly reduced. However, no statistically significant differences were observed in lung septal thickening or hepatic hemozoin deposition, though qualitative trends suggested milder pathology. These findings indicate that Plasmodium anti-GPI scFv is a promising molecule for cerebral malaria intervention, particularly for brain and kidney protection, but further studies are needed to fully characterize its effects on all organ systems, understand its mechanism of action, optimize dosing regimens, and evaluate its pharmacokinetics and long-term safety.

## 6. Patents

This work has a patent application entitled “Anti-GPI single-chain recombinant antibody fragment (scFv) and its use for the treatment of cerebral malaria,” registered under number BR 10 2025 000141 1 at the National Institute of Industrial Property (INPI) in Brazil.

## Data Availability

The data presented in this study are available on request from the corresponding author. The data are not publicly available due to intellectual property considerations and a pending patent application.

## Supplementary Materials

The following supporting information can be downloaded at: https://www.mdpi.com/article/10.3390/ijms27072950/s1.

## Abbreviations

The following abbreviations are used in this manuscript:

AKI	Acute Kidney Injury
BBB	Blood–Brain Barrier
dpi	Days Post-Infection
ECM	Experimental Cerebral Malaria
ELISA	Enzyme-Linked Immunosorbent Assay
GPI	Glycosylphosphatidylinositol
H&E	Hematoxylin and Eosin
HCT	Hematocrit
HGB	Hemoglobin
HPLC	High-Performance Liquid Chromatography
Hz	Hemozoin
IFN-γ	Interferon gamma
IL-10	Interleukin-10
IL-12p40	Interleukin-12 subunit p40
IL-1β	Interleukin-1 beta
IL-6	Interleukin-6
MCH	Mean Corpuscular Hemoglobin
MCHC	Mean Corpuscular Hemoglobin Concentration
MCV	Mean Corpuscular Volume
OD	Optical Density
*P. falciparum*	*Plasmodium falciparum*
PBS	Phosphate-Buffered Saline
PbA	*Plasmodium berghei* ANKA
RBC	Red Blood Cell Count
RDW	Red Cell Distribution Width
RMCBS	Rapid and Quantitative Scale of Murine Coma and Behavior
scFv	Single-Chain Variable Fragment
Th1	T-helper 1
TLRs	Toll-like Receptors
TMB	Tetramethylbenzidine
TNF-α	Tumor Necrosis Factor Alpha
WBC	White Blood Cell
