# Supplementary material for: Anti-GPI scFv as a Promising Tool for Intervention Against Cerebral Malaria in Mice
Source: Int J Mol Sci. 2026 Mar 24;27(7):2950. doi: 10.3390/ijms27072950 (PMC13073753; doi:10.3390/ijms27072950)
Supplement: Supplementary file 1 [file ijms-27-02950-s001.zip › ijms-4136216-supplementary.pdf]

Supplementary Figures

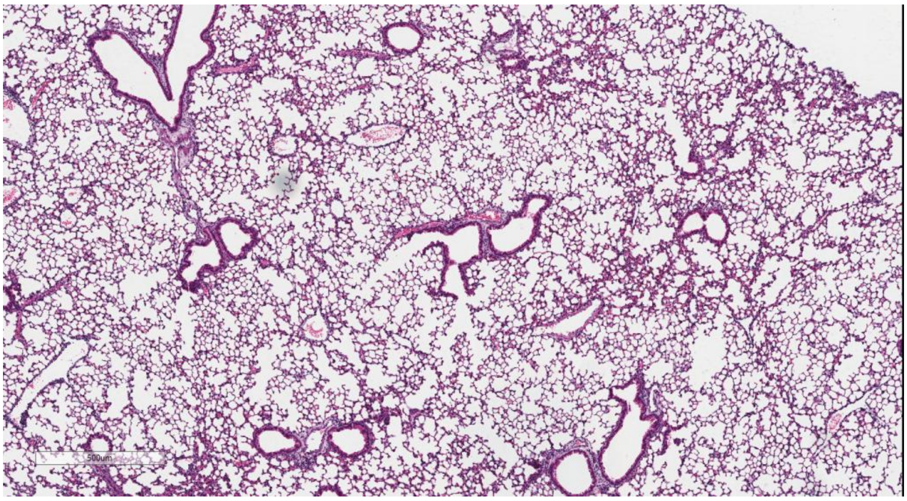

(a)

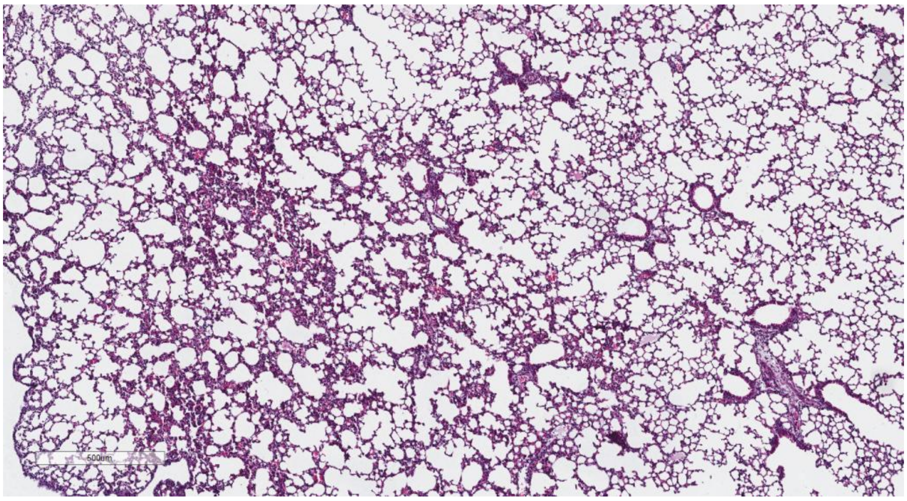

(b)

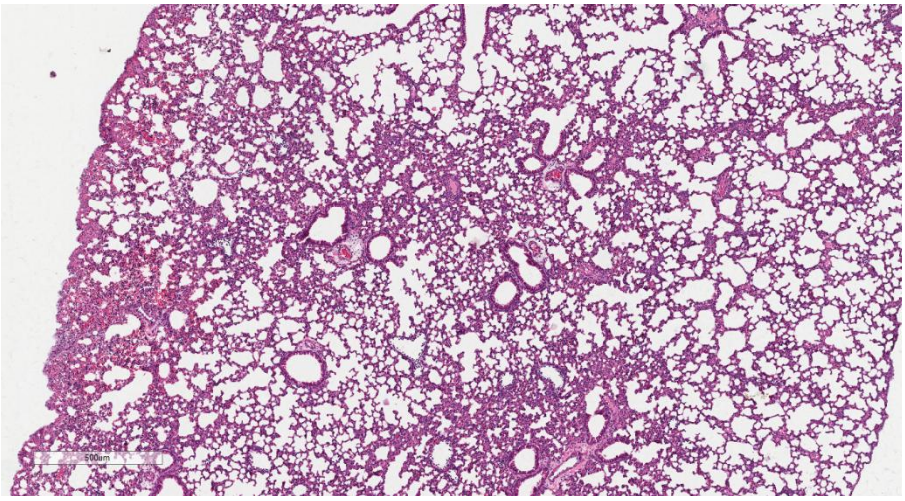

(c)

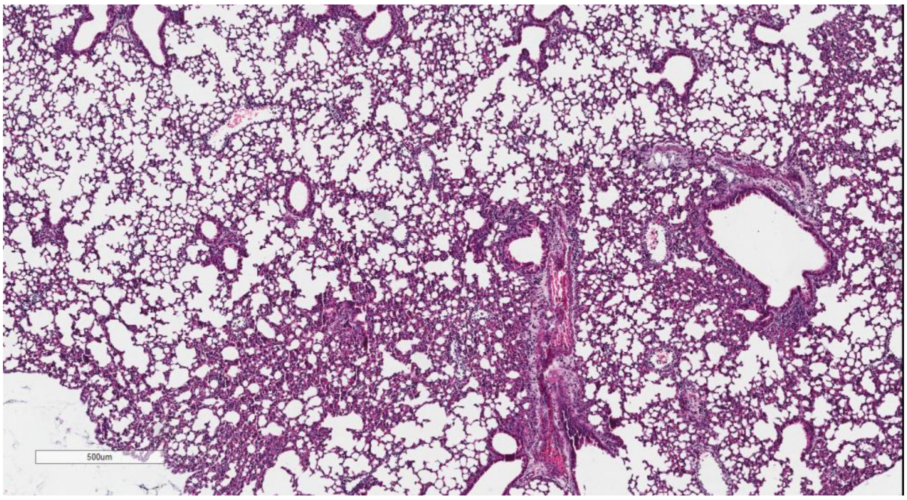

(d)

Supplementary Figure S1. Photomicrographs of the lung demonstrating the presence of thickening of the interalveolar septum. (a) HE- stained lung section showing the pulmonar parenchyma of an uninfected and PBS-treated animal with unchanged lung histology. (b) HE- stained lung section showing the pulmonar parenchyma of an uninfected and scFv-treated animal with mild changed of lung histology, such as, slight thickening of the interalveolar septum (red arrow). (c) PbA-infected and scFv-untreated group showed thickening of the interalveolar septum (red arrow). (d) Histopathological images of the PbA-infected and scFv-treated group, it was observed thickening of the interalveolar septum (red arrow), infiltrates. Tissue samples were harvested from mice euthanized at day 6 post-infection. (a-d), original magnification: x4. Scale bars: 500 μm.

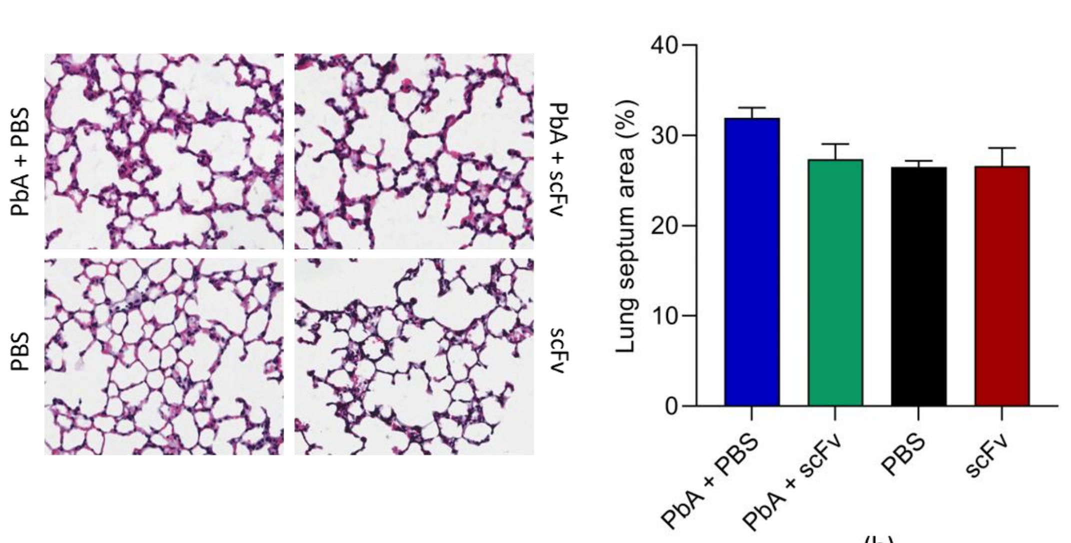

(a) (b)

Supplementary Figure S2. Photomicrographs showing the area of pulmonary septum and a graph quantifying septal thickening. (a) Photomicrographs of the lung demonstrating the presence of inflammatory infiltrate and thickening of the interalveolar septum. (b) Area occupied by the alveolar septum, analyzed using the ImageJ program. Groups: PBS (n=3), scFv (n=3), PbA+PBS (n=6), PbA+scFv (n=6). The graphs represent the mean and standard error of the mean. The representative images are shown at a magnification of 10x, H&E staining. The statistical analysis was conducted using the non-parametric Kruskal-Wallis test followed by Dunns' multiple comparison tests.
